# Complex genetics of female fertility

**DOI:** 10.1038/s41525-018-0068-1

**Published:** 2018-10-12

**Authors:** Rahul Gajbhiye, Jenny N. Fung, Grant W. Montgomery

**Affiliations:** 1 Institute for Molecular Bioscience, University of Queensland, St. Lucia, QLD 4072 Australia; 2 Department of Clinical Research, ICMR-National Institute for Research in Reproductive Health, J. M. Street, Parel Mumbai, 400012 India

## Abstract

Variation in reproductive lifespan and female fertility have implications for health, population size and ageing. Fertility declines well before general signs of menopause and is also adversely affected by common reproductive diseases, including polycystic ovarian syndrome (PCOS) and endometriosis. Understanding the factors that regulate the timing of puberty and menopause, and the relationships with fertility are important for individuals and for policy. Substantial genetic variation exists for common traits associated with reproductive lifespan and for common diseases influencing female fertility. Genetic studies have identified mutations in genes contributing to disorders of reproduction, and in the last ten years, genome-wide association studies (GWAS) have transformed our understanding of common genetic contributions to these complex traits and diseases. These studies have made great progress towards understanding the genetic factors contributing to variation in traits and diseases influencing female fertility. The data emerging from GWAS demonstrate the utility of genetics to explain epidemiological observations, revealing shared biological pathways linking puberty timing, fertility, reproductive ageing and health outcomes. Many variants implicate DNA damage/repair genes in variation in the age at menopause with implications for follicle health and ageing. In addition to the discovery of individual genes and pathways, the increasingly powerful studies on common genetic risk factors help interpret the underlying relationships and direction of causation in the regulation of reproductive lifespan, fertility and related traits.

## Introduction

Female fertility, and the factors that regulate fertility and number of children born are of broad general interest because of their implications for health, population size and ageing. Reproductive life span from the onset of puberty, age-specific fertility rates, and twinning frequency all contribute to fertility.^1–4^ Other factors influencing female fertility include developmental programming, common diseases such as polycystic ovarian syndrome (PCOS) and endometriosis, and the cumulative effects of environmental exposures and lifestyle.^5–8^

**Fig. 1:**
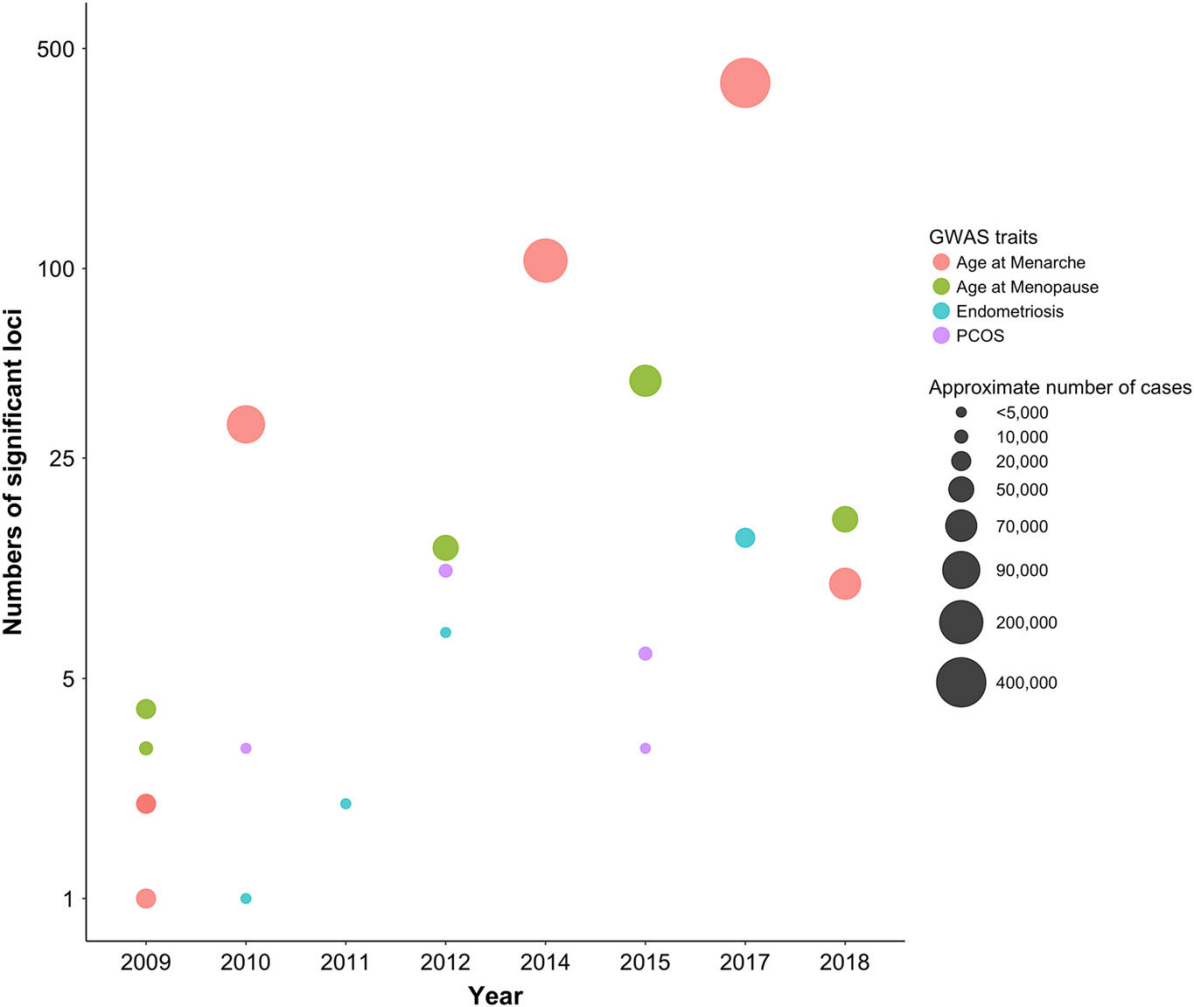
The number of significant association results for GWAS studies for reproductive traits (*Y*-axis—note the log scale) plotted as a function of the date of publication demonstrating progress in GWAS as increased sample sizes and improved genotyping arrays have increased the power of these studies to identity the large number of genetic factors contributing to variation in reproductive lifespan and reproductive diseases

There is substantial genetic variation for common traits associated with reproductive lifespan and common diseases influencing female fertility.^9–12^ This genetic contribution to reproductive traits and diseases can result from rare mutations in specific genes and common variation at many sites in the genome each with small effects. In the last decade, genome-wide association studies (GWAS) have transformed our understanding of genetic contributions to these complex traits and diseases.^13^ The results of these studies have led to discovery of novel genes and pathways influencing specific traits and diseases, new discoveries in disease epidemiology, and the discovery or repurposing of candidate therapeutics.^13^ Results for GWAS studies for reproductive traits (Fig. 1) demonstrate how increases in sample size over time have improved the power of these studies to identity the many genetic factors with small effects contributing to variation in reproductive lifespan and disease.

Studies on genetic contributions to fertility are taking place during a period of considerable demographic change with a substantial fall in age at menarche and a tendency for women to delay childbearing in many countries. This delay in childbearing is associated with an increase in age at first birth, a decrease in the fertility rate because of age-specific effects,^14^ and an increase in the dizygotic twinning rate.^15,16^ Differences in reproductive lifespan are also associated with a range of disease outcomes.^11,17^ Detailed investigation of the genetic basis of the female fertility is providing crucial information for understanding variation in female fertility and preventing or treating disorders that contribute to reduced fertility. This review aims to describe the complexity involved with genetics of female fertility and highlight the important observations emerging from the genetic studies investigating reproductive lifespan, fertility traits, menstrual disorders, and the pathophysiology of disease.

## Genetics of reproductive lifespan

**Fig. 2:**
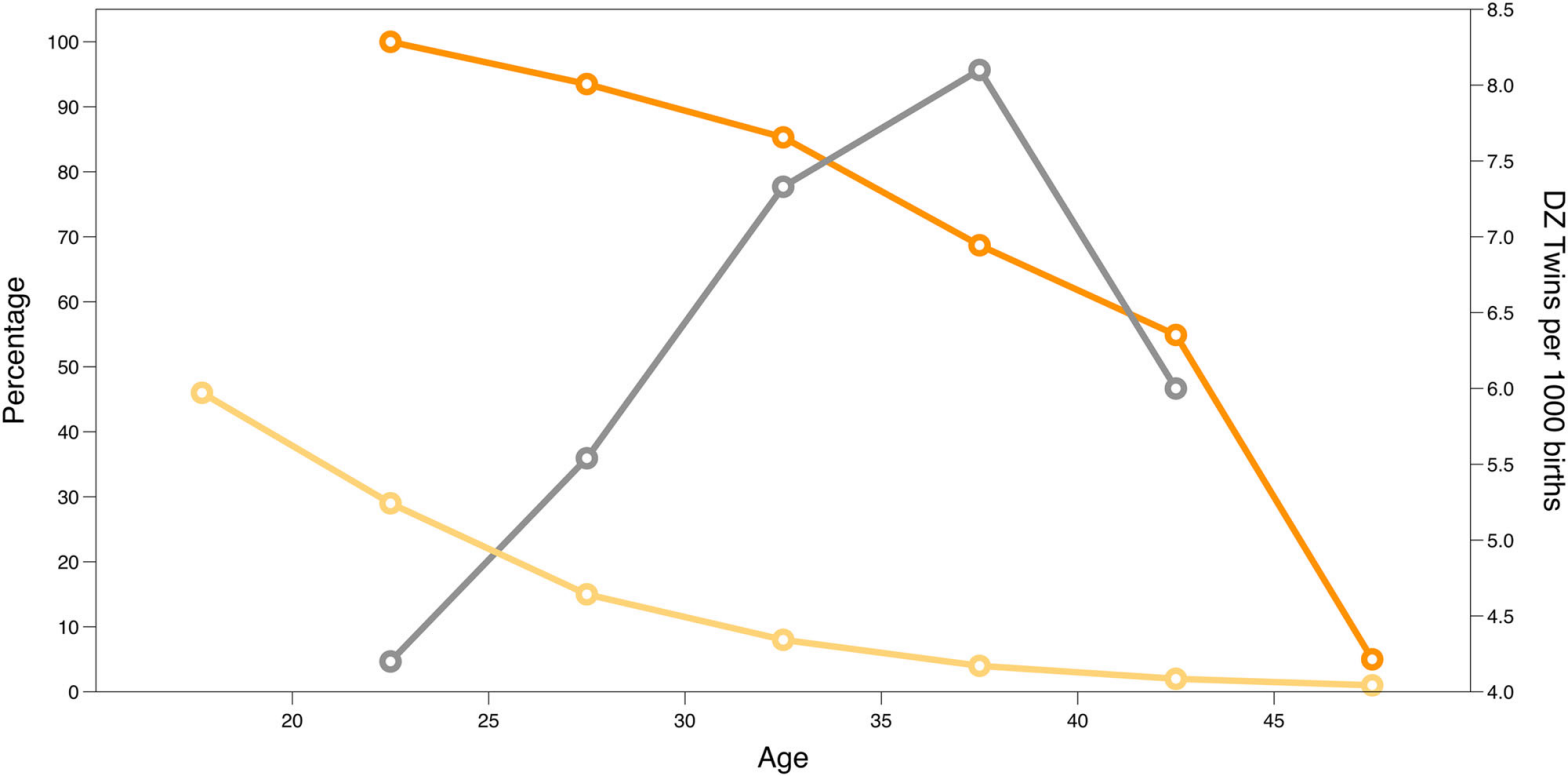
The association between age and fertility (measured as the index of mean fertility rate by age classes^102^ with the fertility rate for the age class 20–24 set to 100%—dark orange line), ovarian reserve (percentage of ovarian reserve remaining at each age^103^ with 100% taken as the maximum ovarian reserve occurring at 18–22 weeks post-conception – pale orange line), and dizygotic twinning rate (dizygotic twins per 1000 births^104^—grey line)

Reproductive lifespan in women is defined as the time from the onset of puberty until the menopause when the pool of oocytes is depleted and menstrual cycles cease.^11^ Primordial follicles develop during gestation and the maximum oocyte pool at birth then declines until exhausted at the time of the menopause, associated with declining fertility and increased twinning rates with age (Fig. 2). There is substantial variation in the timing of these events and in the age at menarche and menopause with impacts on social, health and economic outcomes.^18–21^ The health outcomes from variation in timing of both age at menarche and natural menopause include effects on fertility, cardiovascular disorders, hypertension, glucose intolerance, osteoporosis, obesity, breast cancer, ovarian cancer and endometrial cancer.^11,17^ However, the underlying mechanisms explaining the association of age at menarche and menopause with many of these long-term health impacts are yet to be identified.

### Menarche

Menarche occurs with maturation of the reproductive endocrine system and denotes the onset of menstrual cycles and sexual maturity for women. It usually occurs between 9 and 14 years of age. The average age at menarche has declined over the last century in high income countries including Europe and North America.^22^ Early menarche is associated with early initiation of sexual activity, early pregnancy, high risk of sexually transmitted infections, increased risk for obesity, type 2 diabetes (T2D), breast cancer and cardio metabolic disease.^23–28^ At the other end of the distribution, delayed puberty is associated with short stature and lower bone mineral density.^29^

The timing of puberty varies between individuals and is a highly polygenic trait with both rare and common variants contributing to the variation in age at menarche (AAM). Rare mutations in genes that disrupt the development and function of the gonadotrophin-releasing hormone (GnRH) pathway, pituitary hormones, and their receptors can result in idiopathic hypogonadotropic hypogonadism (IHH) and the absence of puberty.^11,30^ These include mutations in genes for kisspeptin (*KISS1*), GnRH (*GNRH1*), follicle-stimulating hormone (*FSH*), luteinizing hormone (*LH*) and their respective receptors. Some rare mutations for IHH have been implicated in less severe delayed puberty and may contribute to the population variability in AAM.^11^ In contrast, activating mutations in *KISS1* and kisspeptin receptor (*KISS1R*), and paternally inherited mutations in two imprinted genes Makorin ring-finger 3 (*MKRN3*) and Delta-like 1 homologue (*DLK1*) can result in central precocious puberty (CPP).^31,32^ *MKRN3* is an intronless gene located on the long arm of chromosome 15 in a cluster of imprinted genes in the region associated with Prader–Willi syndrome.^31^ *MKRN3* is expressed from the paternal allele (maternally imprinted) in the arcuate nucleus and inhibits the secretion of GnRH during the prepubertal period in childhood.^33^ Mutations associated with CPP^31^ include a four-base deletion in the proximal promoter of *MKRN3* identified in a girl with non-familial idiopathic CPP who exhibited pubertal development around the age of seven.^34^ The deletion was associated with reduced promoter activity in vitro.^34^ *DLK1* is located on chromosome 14 and encodes a transmembrane protein with multiple epidermal growth factor repeats. Like *MKRN3*, *DLK1* is expressed from the paternal allele and paternal inheritance of a complex genomic rearrangement including the translation start site of DLK1 was associated with isolated familial CPP.^32^ The association of CPP with loss-of-function alleles in two paternally expressed genes supports a role for imprinting affecting the timing of puberty.^32^

GWAS of AAM in healthy women in European and Non-European populations have identified key genetic factors regulating puberty timing as reviewed previously.^11,29,30^ The Lin-28 Homologue 28 (*LIN28B*) at 6q21 was the first of the many loci identified from five independent GWAS for AAM.^35^ The most recent GWAS conducted in ∼370,000 women of European ancestry identified 389 independent signals explaining ∼7.4% of the population variance in age at menarche (Fig. 1), corresponding to ∼25% of the estimated heritability.^36^ Eight of the lead SNPs from the GWAS results were non-synonymous variants altering expected protein sequences and lead SNPs were highly correlated with non-synonymous variants implicating a further 24 genes.^36^ These included aromatase (*CYP2C19A1*), and genes disrupted in rare disorders of puberty *GNRH1* and *KISS1*.^36^ Analyses of the data sets for genetic effects on gene expression demonstrated positive enrichment for AAM-associated variants in five central nervous system tissues, notably the pituitary gland and hypothalamus^36^ supporting an important role of central mechanisms in reactivation of the hypothalamic pituitary axis and onset of puberty. Expression of 205 genes in these tissues were regulated by AAM-associated variants including higher expression of *LIN28B* in the pituitary gland associated with later AAM.^36^ Formal analysis of overlap in signals for AAM and genetic effects on gene expression in blood (expression quantitative trait loci; eQTLs) identified evidence for causal or pleotropic effects for 60 regions^36^ with the variant(s) associated with AAM driving variation of a specific genes in each region.

In Japanese women, the mean age at menarche was 13.9 years with a strong secular trend for decreasing age at menarche to a mean age of 12.3 years for women born after 1965.^37^ GWAS in 67,029 women identified 10 significant association signals for age AAM, including signals in two novel regions not reported previously in European women and one novel Japanese-specific signal in a previously reported region near *PTPRD*. More variants had larger effects on early, compared with late menarche timing, consistent with evidence in European studies.^37^

### Menopause

Menopause is defined as the permanent cessation of menstruation resulting from the loss of follicular activity. Menopause generally occurs between ages of 40–60 years with the average age of ~51 years in western countries.^38^ The age at menopause (ANM) has a strong genetic component with more than 50% of variation due to genetic factors.^39^ A genomic analysis for age at natural menopause was conducted in ~70,000 women (Fig. 1) to identify both common and low-frequency variants contributing to genetic variation.^40^ In the analysis of common variants, 54 independent signals were identified in 44 genomic regions (*P* < 5 × 10^−8^) with a range of effect sizes from 0.07 to 0.88 years per allele. Exome array analysis identified genome-wide significant evidence for association with two correlated low-frequency missense variants in DNA helicase B (*HELB*). Although interpretation of the GWAS results have limitations because specific target genes in each region are yet to be determined, the results implicate a substantial role for DNA damage repair in reproductive ageing^40^ including repair of double strand breaks, mismatch repair and base-excision repair. Pathway analysis also suggested enrichment for a set of genes associated with POI, including *MCM8*, *POLG* and *MSH5*.^40^

The risk loci for age at menopause identified in European populations have also been studied in women with different ethnic origins. Out of 22 SNPs identified in European populations, 8 SNPs were reported to be significantly associated with age at menopause in a Chinese population confirming risk SNPs in *NLRP11*, *TMEM150B* and *BRSK1*.^41^ A recent GWAS study for ANM in 43,861 Japanese women identified 16 independent genome-wide significant signals (Fig. 1), of which 8 were novel and not previously reported in Europeans.^37^ Of the remaining 44 significant SNPs reported in European populations and polymorphic in the Japanese population, all had a consistent direction of effect and about half were associated with ANM at nominal levels of significance. Four of the novel signals were highly correlated with missense variants and implicate the genes *GNRH1*, *HMCES*, *ZCCHC2* and *ZNF518A* in the regulation of menopause timing.^37^ The signal in *GNRH1* is the same predicted deleterious missense variant reported for ANM in Europeans.^37^

In African American women, only one of 37 SNPs chosen for replication for age at menarche and none of 16 SNPs for age at natural menopause replicated in the Women’s Circle of Health Study.^9^ No variants met the generally accepted threshold for genome-wide significance. Larger studies will be required to determine whether lack of replication is due to differences in genetic architecture or mechanisms regulating reproductive lifespan in African American women.

### Potential health impacts and links with other health disorders

Variation in AAM and ANM have wide-ranging effects on human health. In a study in the UK Biobank, age at menarche was associated with 26 adverse health outcomes.^14^ Earlier AAM is associated with higher risks for type 2 diabetes (T2D), cardiovascular disease, and effects on gynaecological, gastrointestinal, musculoskeletal, and respiratory conditions.^14^ The relationships are complex with non-linear relationships between AAM and T2D and cardiovascular disease.^11,14,42^ Early menarche increases risk for both T2D and cardiovascular disease while later menarche also increases risk for cardiovascular disease, but is neutral for T2D.^11^ BMI also plays a role for many of the same health outcomes. Following adjustment for body composition and socio-economic status, associations with early menarche remained significant for 14 health outcomes and associations with late menarche were significant for eight health outcomes.^14^ Early menarche was associated with higher risks for uterine fibroids, endometriosis and earlier natural menopause.

Later age at menopause is associated with increased risks for breast, ovarian and endometrial cancer. Many of the genetic markers influencing menopause are related to DNA damage repair genes including *BRCA1*, *MSH6* and *CHEK2* that also predispose to familial cancers.^39^ In addition, later age at menopause results in longer exposes to high levels of oestrogen for women. Poor DNA damage response that increases cancer risk might be expected to lead to earlier menopause, opposite to the evidence from epidemiological studies. Using the available genetic data from studies for breast cancer and age at menarche, predicted age at menopause from genetic variants showed increased risks of breast cancer with later age at menopause^40^ and the effects were greater in oestrogen receptor positive breast cancer. Genetic variants associated with DNA damage repair genes had smaller effects than other markers.^40^ Taken together, the results suggest that increased exposure to oestrogen with increased reproductive lifespan is the predominant effect on risk for breast cancer.

## Genetics of fertility and reproductive behaviour

Fertility traits in human populations are under genetic control.^43^ Successful reproduction denoted by age at first birth (AFB) and the associated behaviour of age at first sexual intercourse (AFS) are both moderately heritable and genetically correlated.^44^ Estimates from the UK Biobank for SNP-based heritability for AFS and AFB were 0.242 (s.e. = 0.010) and 0.290 (s.e. = 0.015) respectively. There was a strong genetic correlation between AFS and AFB (*r*_g_ = 0.86) and moderate genetic correlations between menarche and both AFS (*r*_g_ = 0.22) and AFB (*r*_g_ = 0.24).^44^ GWAS identified 34 genome-wide significant signals associated with AFS in women with replication in deCODE data for the Icelandic population, and in the Women’s Genome Health Study. The signals include association with intronic SNPs in the oestrogen receptor 1 (*ESR1*) gene that are also associated with AFB and with the number of children ever born. The *ESR1* SNPs associated with AFB are unrelated to SNPs at this locus associated with puberty timing and breast cancer. On chromosome 3, the SNP rs2188151 associated with AFS is highly correlated with a missense variant in the semaphorin protein *SEMA3F*. The SNP also influences expression (is a *cis*-eQTL) for the RNA binding protein *RMB6*. The AFS decreasing allele is associated with later age at menarche, earlier AFB and greater numbers of children born.^44^

A large study of genetic effects on reproductive behaviour in 62 cohorts of European ancestry (>250,000 individuals) identified 10 independent genomic loci associated with AFB in women, men or both.^45^ Follow-up analyses identified a number of genes in the genomic regions associated with AFB and number of children born that could be prioritised for functional studies. The critical SNPs on chromosome 1 associated with AFB and number of children ever born are correlated with likely functional nonsynonymous SNPs in two genes; the CREB-regulated transcription co-activator 2 (*CRTC2*) which acts downstream of FSH in ovarian granulosa cells, and CREB protein 3 like 4 (*CREB3L4*) which is highly expressed in reproductive tissues in both females and males.^45^ The lead SNP (rs2777888) for AFB on chromosome 3 is associated with altered DNA methylation or expression of several genes with a role in cell cycle progression and/or sperm function.^45^ Further functional experiments will be necessary to determine the specific genes and mechanisms of action of the large number of genetic variants influencing these important traits.

## Ovarian function and dizygotic twinning

Ovarian reserve is one of the crucial elements of female fertility (Fig. 2) and several GWAS report genetic associations and pathways responsible for reproductive aging and POI.^46^ POI is generally defined as the onset of menopause in women under the age of 40 years. Genetic mutations have been reported in a number of candidate genes, although many reported results have not been replicated.^47^ There is good evidence for deleterious effects of mutations in Bone Morphogenetic Protein 15 (*BMP15)*, Progesterone receptor membrane component 1 (*PGRMC1*) and the pre-mutation in the Fragile-X mental retardation 1 (*FMR1*) locus on the X chromosome.^47^ Mutations in other genes, present in low frequencies in some populations, are likely to influence POI including *GDF9*, Folliculogenesis specific bHLH transcription factor (*FIGLA*), and Newborn ovary homeobox gene (NOBOX).^47^ The 5-prime untranslated region of the FMR1 gene contains a CGC repeat that varies in length and expansion of the repeat to >200 copies causes Fragile-X syndrome. Repeat expansion to the pre-mutation range is associated with premature menopause, but there is no influence of shorter repeat lengths in the normal range or the longer repeats that cause Fragile-X syndrome.^48^

A total of six GWAS have been conducted to identify the risk loci in POI in different populations as reviewed in detail elsewhere.^47^ Although GWAS have identified multiple loci associated with POI in Chinese, Korean, and Dutch women,^47^ the small samples studied (<1000) have low statistical power and none identified signals exceeding 10^−6^. Pathway analysis of suggestive GWAS loci for ANM do show enrichment for known POI genes.^39^ There are ~80 gene disorders influencing extremes of reproductive function. The genes are involved in diverse biological processes, including control of the cell cycle, DNA damage response and repair, hormone signalling and gonadal development. Genetic variants in or near many of these genes have variable effects from very early menopause to alterations of just a few weeks.^39^ Future studies involving larger data sets and meta-analysis of combined GWAS will be necessary to identify risk loci associated with POF.

The spontaneous dizygotic (DZ) twinning rate is associated with fertility and comparisons with the frequency of monozygotic twins provide a useful index of fertility in a population.^49^ DZ twins arise from the ovulation of two follicles after a complex process of follicle growth, selection and ovulation. The frequency of DZ twinning ranges from 6–15 per 1000 live births and varies with maternal age and ethnicity.^50^ DZ twinning is influenced by genetic factors and the DZ twinning rate for sisters of women with spontaneous DZ twins is ~2.5 times higher than the twinning rate in the general population.^50^ Direct evidence for genetic variants influencing rates of DZ twinning were first identified in animal studies. Domestic sheep generally have 1–2 offspring at each pregnancy. Linkage and positional cloning identified mutations in autosomal and X-linked genes that increased ovulation and twinning rates in heterozygous carriers.^51–53^ The mutations responsible were identified in the genes for Bone Morphogenetic Protein Receptor Type 1B (*BMPR1B*) and *BMP15*, respectively; a receptor and ligand in the ovarian signalling pathway for BMP15.^51,53^ Multiple mutations have now been reported in both *BMP15* and the closely related signalling molecule growth differentiation factor 9 (*GDF9*).^54^ Physiological and genotype-phenotype studies of strains carrying the different mutations demonstrate the importance of BMP signalling, and the balance between BMP15 and GDF9 in follicle survival, maturation, and control of ovulation.^54^

Some mutations in *BMP15* and *GDF9* have alternative phenotypes depending on whether they are heterozygous or homozygous (carried as one or two copies respectively). Increased ovulation rates occur in heterozygous carriers of the mutations as described above. In contrast, homozygous carriers with two copies of the loss-of-function mutations in *BMP15* and *GDF9* in sheep have streak ovaries and are completely infertile.^54^ These genes are candidates for infertility in women and studies in women with primary ovarian insufficiency (POI), also known as premature ovarian failure (POF), have identified mutations in both genes associated with POI.^55^ Confirmation through segregation studies is difficult in families with infertility disorders, but molecular and functional studies for several of these variants suggest loss-of-function effects consistent with the view that two functional copies of BMP15 are required for an adequate ovarian reserve in women.^55^ Low-frequency variants in *GDF9* are associated with increased risk for DZ twinning in women heterozygous for these variants.^56^ Similar studies found no evidence that rare and low-frequency variants in *BMP15* influence DZ twinning.^57^ Mutation screening of *BMPR1B* in mothers of DZ twins identified a coding variant (p.Gln294Glu) altering the same amino acid as a sheep *BMPR1B* functional variant.^58^ This mutation was not seen in other families and is of unknown significance.

Rare and low-frequency variants account for only a small proportion of variation in DZ twinning.^56^ In a search for common genetic variants influencing DZ twinning, a GWAS in 1980 mothers of spontaneous DZ twins and 12,953 controls identified significant association with DZ twinning for SNPs close to Follicle-Stimulating Hormone Beta Subunit (*FSHB*) and SMAD Family Member 3 (*SMAD3*).^59^ The risk alleles for the SNPs close to *FSHB* and *SMAD3* increased the frequency of twin births in the Icelandic population by 18 and 9%, respectively. The lead SNP associated with DZ twinning on chromosome 15 maps to the first intron of *SMAD3*, strongly expressed in the human ovary, where it promotes granulosa cell proliferation and steroidogenesis.^59^ The region of chromosome 15q22.33 also includes SMAD Family Member 6 (*SMAD6*). A major gene increasing ovulation rate and twinning in cattle maps to the equivalent genomic region on bovine chromosome 10 (the location of both *SMAD3* and *SMAD6*).^60^ Recent analysis of gene expression in granulosa cells from carriers and non-carriers of the cattle gene demonstrated a six-fold increase in expression of *SMAD6* in gene carriers.^61^ SMAD6 is an inhibitor of BMP/SMAD signalling and over-expression of *SMAD6* is consistent with loss-of-function mutations in BMP signalling increasing ovulation rate in sheep. Further research is required to determine whether variants associated with DZ twinning on chromosome 15 act through effects on *SMAD3*, *SMAD6* or some other mechanism.

## Diseases influencing fertility

### Polycystic ovary syndrome

Polycystic ovary syndrome (PCOS) is a complex, hormonal and metabolic disorder affecting 5–20% of women of reproductive age globally and characterised by hyperandrogenism, ovulatory dysfunction, polycystic ovarian morphology and gonadotropic abnormalities.^62,63^ PCOS is the most common cause of infertility^64^ and also increases the risk for type 2 diabetes, gestational diabetes, venous thromboembolism, cerebrovascular and cardiovascular disease and endometrial adenocarcinoma.^65^ The aetiology of PCOS remains unclear with diagnostic criteria proposed for PCOS^66^ including the National Institutes of Health (NIH), Rotterdam and Androgen Excess Society (AES) criteria. Familial aggregation and twin studies suggest genetic factors play a strong role in pathogenesis of PCOS with heritability estimates of 70%.^65,66^

The first GWAS conducted in Chinese patients in 2011 (Fig. 1) identified three genomic regions associated with the disease.^67^ Additional studies^66,68,69^ identify 16 independent signals in 15 genomic regions associated with PCOS including signals near important reproductive hormone genes *FSHR*, *LHCGR* and *FSHB*.^70^ The signals also include variants in or near three epidermal growth factor genes and genes associated with diabetes.^40,70^ As with similar studies in other reproductive diseases, the total heritability explained by GWAS identified PCOS risk SNPs is relatively low (<10%).^65^

Mendelian randomisation is an analytical method using genetic variation to investigate the likely causal relationship between an exposure trait (or risk factor) and a health outcome. Genetic variants are inherited independently and fixed at birth and subject to less confounding than other measured risk factors. Increasing evidence of association between genetic variants and many common traits means Mendelian randomisation models can use genetic variants associated with a risk factor to infer relationships with a health outcome. The models assume the genetic variants used have strong evidence for association with the risk factor and do not influence the outcome through other unrelated biological pathways. In PCOS studies, Mendelian randomisation demonstrated causal roles for higher BMI, greater insulin resistance and reduced sex hormone binding globulin concentrations in serum.^68^ The causal role of PCOS risk SNPs for higher BMI, higher insulin resistance, and lower levels of sex hormone binding globulin (SHBG) has direct clinical applications for planning lifestyle modification as a prevention strategy and inclusion of metformin in treatment plans for PCOS.^68^ Discovery of additional genetic factors and further characterisation of the signals identified will provide greater insight into the pathogenesis of the complex phenotypes in PCOS.

### Endometriosis

Endometriosis is a complex disease characterised by ectopic lesions of tissue resembling endometrium in the peritoneal cavity.^7^ The disease affects 7–10% of women and is associated with pain and infertility. Early twin studies identified evidence for genetic effects on the liability for hysterectomy,^71^ one of the most commonly performed surgical procedures for women. Major indications for hysterectomies in reproductive age women are endometriosis (30%) and uterine leiomyomas (>50%).^72,73^ Subsequent studies in twins on genetic influences on the liability for endometriosis estimated the heritability at ~50%.^74,75^

Genomic regions and genes associated with endometriosis risk are reviewed in detail elsewhere.^76–78^ The most recent meta-analysis (Fig. 1) identified 14 genomic regions associated with disease risk.^79^ Endometriosis is an oestrogen-dependent disease and Oestrogen receptor 1 (*ESR1*) is the predominant receptor for oestrogen action in the endometrium.^80^ Genomic signals associated with endometriosis include regions flanking the gene for *ESR1*, signals upstream of follicle-stimulating hormone beta subunit (*FSHB*) known to increase FSH concentrations,^79^ and near the oestrogen-regulated and early response gene (*GREB1*) first identified in breast cancer cell lines and tumours.^81^ Other regions include candidate genes with roles in cell migration, adhesion and proliferation including Cell Division Cycle 42 (*CDC42*), Cyclin-Dependent Kinase Inhibitor 2B Antisense (*CDKN2B-AS1*) and Kinase insert domain receptor (*KDR*).^82–84^

Mechanisms leading to formation of lesions are poorly understand, but one source for cells initiating these lesions is thought to be cells shed from the endometrium and deposited in the pelvic cavity through retrograde menstruation.^7,76,85^ On chromosome 1, studies on genetic regulation of gene expression in blood^86^ and endometrium^78,86^ show the critical SNPs in this region influence expression of both the long non-coding RNA *LINC00339* and *CDC42*. There are chromatin interactions between risk SNPs and gene promoters for both *LINC00339* and *CDC42*.^86^ Luciferase reporter assays support the effect of genetic differences on the interaction between the regulatory element and the promoter of *CDC42*.^86^ Formal analysis of the overlap of signals for endometriosis risk and genetic effects on gene expression provides strong evidence that key SNPs associated with endometriosis on chromosome 1 and chromosome 12 regulate *LINC00339* and vezatin *VEZT* expression, respectively.^78,87^

## Genetic architecture of reproductive lifespan and fertility

Genetic studies discussed above demonstrate the complex variation contributing to the timing of puberty, menopause, ovarian function and twinning. Genetic contributions include the effects of multiple common variants with small effects on reproductive traits and disease, and rare variants with large effects contributing to failures in development, precocious puberty, delayed puberty, premature ovarian failure and increased twinning. In some cases (Tachykinin Receptor 3; *TACR3* and age at menopause), rare, low-frequency and common variants all influence the same trait.^11^ In other examples (*SOX10*, *CHD7*, *FGFR1*, *KISS1R*, and *TAC3*), rare variants cause hypogonadotropic hypogonadism while common variants influence age at menopause.^11^ These differences, and evidence for the different effects on ovulation rate and streak ovaries for heterozygous and homozygous mutations in BMP15 and GDF9 in sheep^54^ show disruption of gene function or altered regulation of the same genes can have different effects on reproductive traits and diseases.

**Fig. 3:**
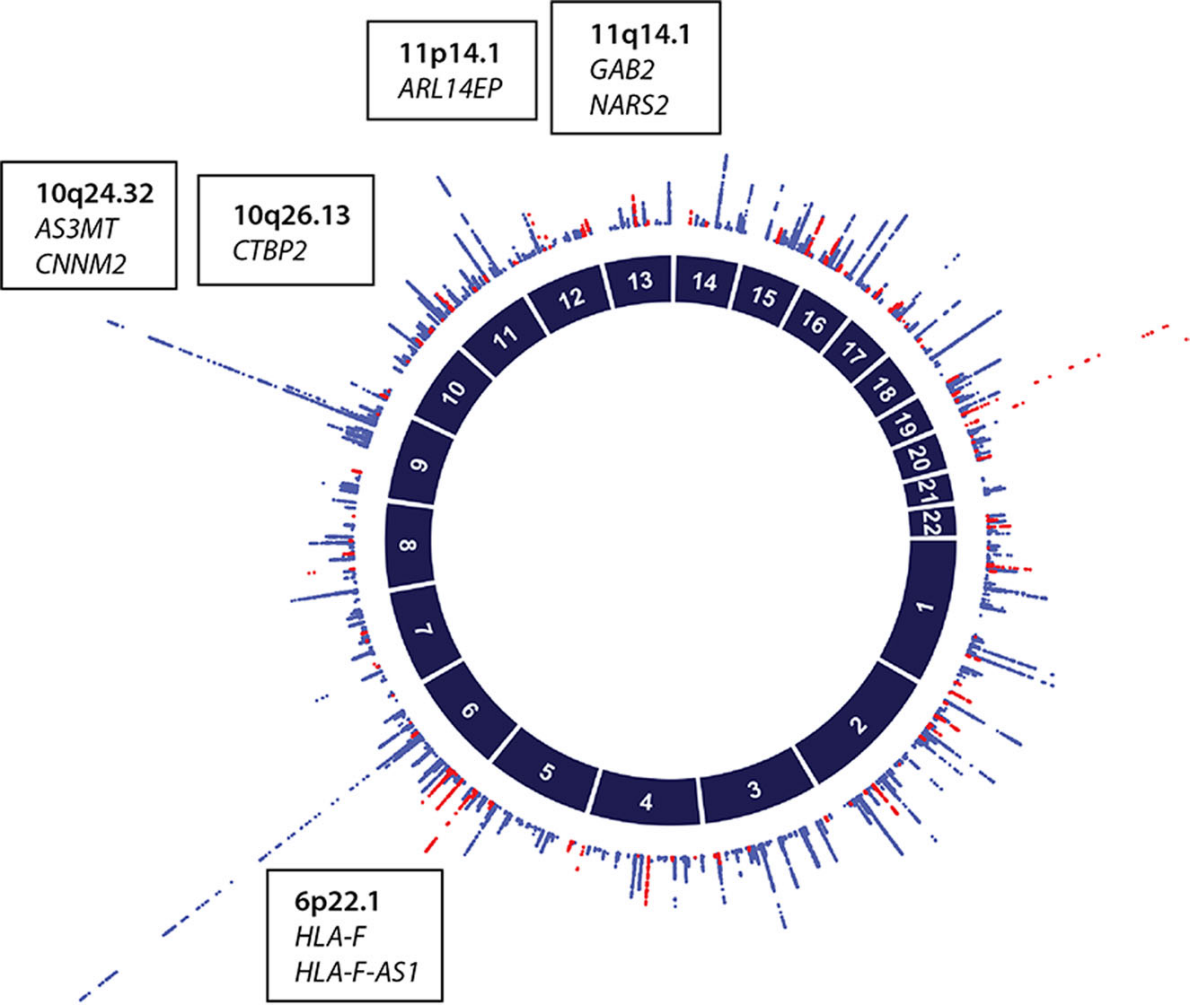
Circle plot showing chromosome number (dark blue, inner circle). Results of association between individual SNPs with Age at Menarche^36^ and Age at Menopause^40^ are plotted as –log10 (*P* values) (blue or red, outer circle). The red dots represent the associations with Age of Menopause above a threshold of *p* *<* 1 × 10^−5^ and the blue dots represent the associations with Age of Menarche above a threshold of *p* *<* 1 × 10^−5^. Genomic regions, where SNPs for Age at Menarche at genome-wide significance (*p* *<* 5 × 10^−8^) overlap with the genomic regions where SNPs for the Age at Menopause have suggestive evidence for association (*p* < 5 × 10^−6^) are identified on the figure within the text boxes including the chromosome region and nearby biological candidate genes

Epidemiological studies suggest a relationship between early menarche and early menopause. However, the recent genetic studies show this relationship is more complex.^11,39^ Studies in the UK Biobank show earlier menopause is associated with both early and later age at menarche.^14^ Genetic marker studies for common risk factors influencing AAM, ANM and common reproductive diseases define genomic regions that may include many genes.^12^ Functional effects may act through one of several genes and inferences based on the best candidate genes in each region should be treated with caution until appropriate functional studies define the causal genes. Nevertheless, the GWAS studies for AAM and ANM show limited overlap in the genomic regions associated with the two traits (Fig. 3) and implicate different pathways influencing age at menarche and menopause with likely candidates for AAM implicating gene regulation in the hypothalamus and pituitary gland^36^ while candidates for ANM implicated DNA damage repair.^40^

## Overlap in genetic contributions to different traits and diseases

**Fig. 4:**
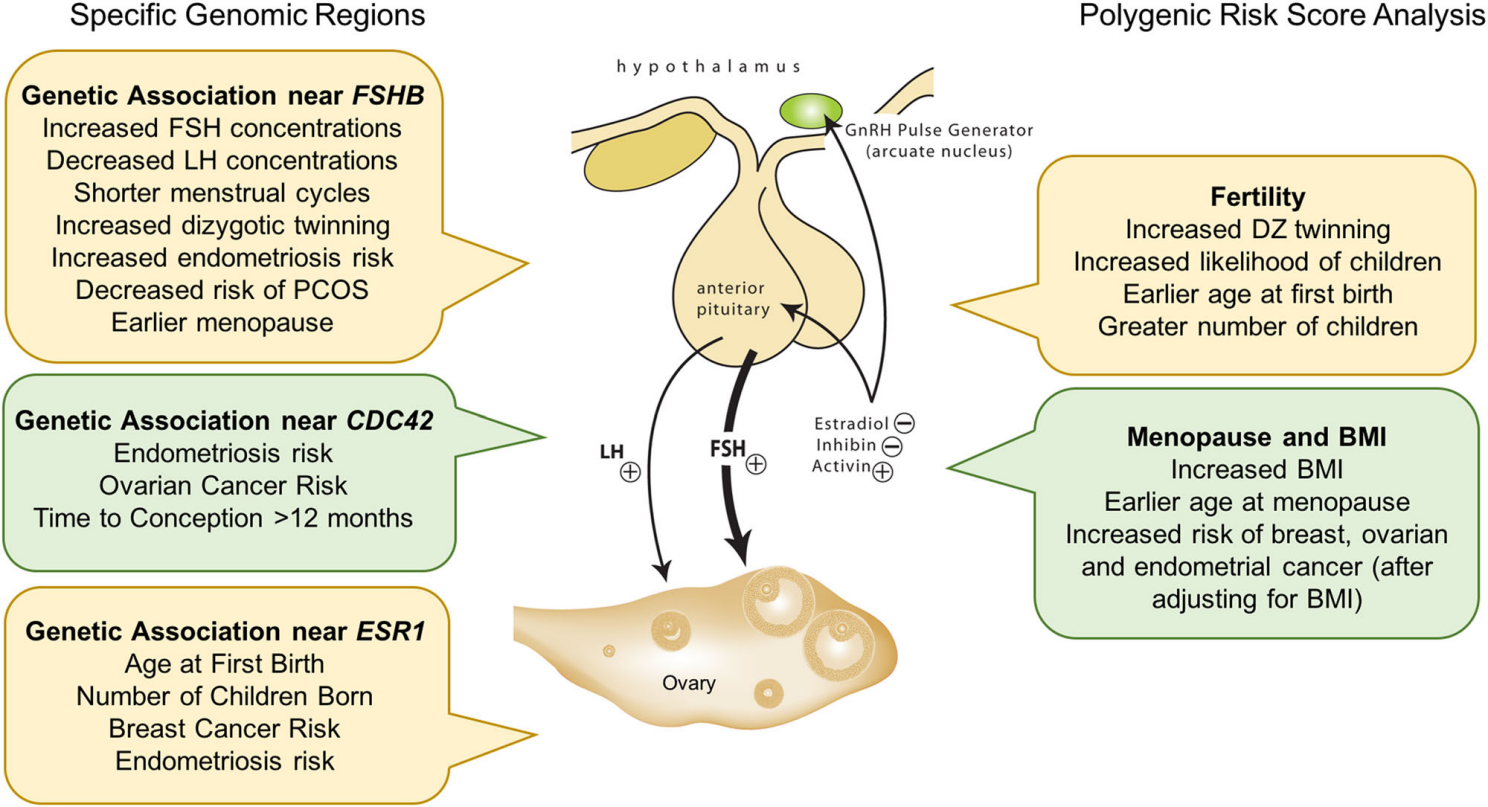
Genetic studies reveal several genomic regions with strong associations for multiple reproductive traits with three examples shown on the left-hand side of the figure. The Polygenic risk score (PRS) combines association results from genome-wide genotyping into a single estimate of the genetic risk for a disease or trait and is calculated from the number of risk alleles carried by an individual, weighted by the effect size estimated from the discovery sample. The polygenic risk scores calculated from the results of large GWAS data provide insights into shared genetic risk between traits and help to understand the complex relationships between related traits with two examples shown on the right-hand side of the figure. The examples are redrawn with permission from a figure published in Nature^105^ on age at menarche [Perry, J. R. et al.^105^]

The hypothalamic/pituitary/ovarian axis plays a central role in development and function of many reproductive processes. It is perhaps not surprising that variation affecting key genes in this pathway influence multiple diseases and traits. A notable example is SNPs upstream of the transcription start site of *FSHB*. SNPs in this region are associated with increased concentrations of circulating FSH,^88^ decreased concentrations of LH,^88^ shorter menstrual cycles,^89^ increased dizygotic twinning,^54^ decreased risk of PCOS,^69^ increased endometriosis risk,^95^ and earlier menopause^96^ (Fig. 4). It remains to be determined if the association signals for all these traits act through the same causal SNPs and functional mechanisms. In the study on FSH and LH concentrations, the genetic signals had opposite effects on hormonal concentrations despite positive overall correlations in FSH and LH concentrations supporting complex relationships in the regulation of FSH and LH. Other associations support common mechanisms since higher FSH concentrations at the time of follicle selection are associated with increased DZ twinning^44^ and shorter menstrual cycles are associated with higher endometriosis risk.^90^

Potential overlap for other traits includes genes influencing both DZ twinning and POI and endometriosis and ovarian cancer. There is a small, but significant increase in mothers of DZ twins reaching menopause before the age of 40 compared with mothers of MZ twins.^91^ Low-frequency variants in *GDF9* play a role in both DZ twinning and POI, including at least one *GDF9* variant influencing twinning and seen in a patient with POI.^55^ Genomic loci with common variants influencing age at natural menopause are located close to genes known to carry rare mutations causing hypogonadotropic hypogonadism (*CHD7, FGFR1, SOX10, KISS1R*, and *TAC3*) and genes associated with POI.^40^ Further research will help to understand the relationships between ovarian development, disorders of puberty, DZ twinning, POI, and age at natural menopause.

Understanding the functional consequences of genetic association for the same signals in different traits will provide important insights into the similarities and differences in gene regulation underlying risk for the different diseases. The genetic locus associated with endometriosis risk on chromosome 1p36 (discussed above) overlaps completely with an association signal for ovarian cancer.^79,92^ The association signals are located in *WNT4*.^79,92^ Subsequent follow-up functional studies show correlated association signals for endometriosis in this region regulate *LINC00339* and *CDC42* not *WNT4*,^83^ and one or both target genes may also play a role in ovarian cancer risk. There is genetic association near the oestrogen receptor 1 (*ESR1*) locus with four independent signals for endometriosis and five independent signals for breast cancer^79,93^ (Fig. 4). Analysis showed overlap for only one of the signals, in an intron of *ESR1*.^79^ Intronic variants in *ESR1* are also associated with other reproductive traits, including age at first birth and number of children born.^44^ In agreement with limited overlap between signals for endometriosis and breast cancer in this region, signals for age at first birth and number of children born did not overlap with other disease associations for timing of puberty, breast cancer, breast size, or bone mineral density in the *ESR1* region^44^ suggesting complex regulation of gene expression and disease outcomes at this locus.

In addition to discovery and overlap in effects for individual genes, GWAS data provides powerful approaches to understand shared genetic risk between traits and diseases. Genome-wide SNP genotype data from a discovery sample can be used to estimate the genetic variation due to common SNPs or SNP heritability, and to calculate a polygenic risk score for individuals in an independent sample.^13,94^ The Polygenic risk score (PRS) combines association results from genome-wide genotyping into a single estimate of the genetic risk for a disease or trait for each individual. The PRS score is calculated from the number of risk alleles carried, weighted by the effect size estimated from the discovery sample.^13,94^ It is often standardised to a mean of zero with a standard deviation of 1 for ease of interpretation.^94^ The risk scores are not very informative for individual prediction, but explain sufficient variation to determine individuals at highest or lowest risk in populations, and to understand genetic contributions to related traits.^13^ Where samples are fully independent, calculating genetic contributions to related traits provides a powerful design reducing issues of shared environment and ascertainment in epidemiological studies. Overlap between endometriosis and ovarian cancer extends beyond the observed overlap at the chromosome 1p36 locus discussed above. Observations from epidemiological studies can be confounded due to diagnosis of endometriosis at laparoscopy close to a diagnosis of ovarian cancer. PRS analysis with genotype data from independent samples for endometriosis and ovarian cancer showed shared genetic risk between endometriosis and most histotypes of ovarian cancer^95^ suggesting some common molecular pathways for the two diseases including the overlap at chromosome 1p36. Prediction of individual risk is improving for some diseases with better estimates of the genetic risk factors. While not sufficiently predictive in isolation, risk scores may be used in combination with other clinical data for patient stratification. The application of risk prediction to inform breast cancer screening is being evaluated in the population based Women Informed to Screen Depending On Measures of risk (WISDOM) study.^96^

As noted earlier, there are significant additive genetic effects for age at first birth^45,97^ and number of children ever born.^97^ Results show a significant negative genetic correlation between number of children ever born and the age at first birth suggesting genes contributing to later first birth are associated with fewer children.^97^ These relationships are complex because environmental effects and demographic changes also play a role with women choosing to delay childbearing in many countries. Relationships between age at first birth and lifetime number of children are also associated with genetic effects on twinning. Mothers of twins tend to have an earlier age at first birth and raise more children to adulthood in favourable environments.^98,99^ Polygenic risk score analysis from the GWAS results for DZ twinning suggest genes contributing to DZ twinning may partly explain genetic effects on fertility. The polygenic risk score for DZ twinning was significantly associated with DZ twinning in an independent sample from Iceland.^59^ In this sample, the risk score for DZ twinning was associated with a higher likelihood of having children, earlier age at first birth, and greater number of children.^59^

Genetic studies can also inform complex relationships between reproductive traits and related health outcomes. A large-scale meta-analysis supported an association of early menarche and late menopause with increased risk of breast cancer and demonstrated that excess risk associated with advancing menarche by one year was higher than excess risk associated with lengthening menopause by one year.^100^ This epidemiological evidence is supported by the Mendelian randomisation analyses demonstrating the causal relationship between delayed natural menopause and increased breast cancer risk.^40^ Understanding these relationships is further complicated by effects of variation in body mass index (BMI). There is a strong inverse genetic correlation between AAM and BMI.^36^ Thirty-nine signals overlap between AAM and BMI, but most AAM signals make some contribution to adult BMI.^36^ Using information from different genetic studies allowed estimates of the direct effects of AAM on sex-steroid-sensitive cancers after adjusting for genetically predicted BMI. In these models, later AAM was associated with reduced risks for breast, ovarian and endometrial cancers.^36^ Analysis of cancer subtypes suggests effects were most strongly associated with oestrogen receptor positive breast cancer and serous ovarian cancer. The effects of earlier puberty timing on higher risks of the sex- steroid sensitive cancers might be related to longer duration of exposure to sex steroids and/or increased activity of the hypothalamic-pituitary axis associated with earlier puberty and reproductive traits including dizygotic twinning.

## Summary and conclusions

Recent studies have made great progress towards understanding the genetic factors contributing to variation in traits and diseases influencing female fertility. These genetic studies are providing important insights into the complex biology contributing to female fertility. The data emerging from GWAS demonstrate the utility of genetics to explain epidemiological observations, revealing shared biological pathways linking puberty timing, fertility, reproductive ageing and health outcomes. For example, many variants influencing menopause suggest genes and pathways with known roles in DNA damage/repair with implications for follicle health and ageing, although we do not yet have a full picture of the mechanisms responsible.

The effect sizes for common variants are small and, as emphasised in this and other reviews, large studies are essential to identify a substantial fraction of common variants underlying variation for these traits and diseases. In addition, GWAS identify genomic regions, but not the specific genes and pathways regulating variation in traits and diseases. To date, we lack definitive evidence for the causal variants and target genes for most of these results. Despite these gaps, examination of potential candidate genes in the multiple regions identified through GWAS provide tantalising evidence for common pathways affecting reproductive lifespan and fertility.

The challenge now is to provide functional evidence for the specific genes and pathways regulated by the genetic variation influencing fertility. Multiple lines of evidence from both functional and genomic studies will be required to identify these gene targets and the mechanisms responsible. This is becoming more feasible with advances in genomics, large-scale publically available data, and genome editing.^101^ This in turn will provide much better understanding of the specific pathways regulating individual components of reproductive lifespan and fertility, and the complex interactions between pathways and health outcomes. The knowledge gained will suggest possible interventions and ways to better predict and minimise health impacts of these important life events.

Genetic risk scores from common variants are not able to predict disease risk for individuals. As the estimates improve from more powerful studies, they may be able to predict women at higher or lower risk for specific diseases and this may be useful in some diagnostic settings. Genetic variants associated with natural menopausal timing also influence the menopausal timing in women undergoing radiation or chemotherapy suggesting that in the future, genetic risk prediction models could be useful for counselling related to family planning, lifestyle choices or use of modern techniques for fertility preservation such as oocyte cryopreservation.

Epidemiological evidence for comorbidity between traits can be difficult to interpret because of the issues of sample ascertainment. Genetic risk scores add a valuable method to gain insight into the complex relationships in the overlap between traits and diseases. These analyses will also improve as the risk scores can be derived from increasingly powerful studies to help interpret the underlying relationships and direction of causation in the regulation of reproductive lifespan, fertility and related traits.
